# Sustained Low Relapse Rate With Highly Variable B-Cell Repopulation Dynamics With Extended Rituximab Dosing Intervals in Multiple Sclerosis

**DOI:** 10.1212/NXI.0000000000200056

**Published:** 2022-11-21

**Authors:** Chiara Starvaggi Cucuzza, Elisa Longinetti, Nicolas Ruffin, Björn Evertsson, Ingrid Kockum, Maja Jagodic, Faiez Al Nimer, Thomas Frisell, Fredrik Piehl

**Affiliations:** From the Department of Clinical Neuroscience (C.S.C., E.L., N.R., B.E., I.K., M.J., F.A.N., F.P.), Karolinska Institutet, Stockholm, Sweden; Center for Molecular Medicine (C.S.C., N.R., I.K., M.J., F.A.N., F.P.), Karolinska University Hospital, Stockholm, Sweden; Department of Neurology (B.E., F.P.), Karolinska University Hospital, Stockholm, Sweden; Center for Neurology (C.S.C., I.K., M.J., F.A.N., F.P.), Academic Specialist Center, Stockholm, Sweden; and Clinical Epidemiology Division (T.F.), Department of Medicine Solna, Karolinska Institutet, Stockholm, Sweden.

## Abstract

**Background and Objectives:**

B cell–depleting therapies are highly effective in relapsing-remitting multiple sclerosis (RRMS) but are associated with increased infection risk and blunted humoral vaccination responses. Extension of dosing intervals may mitigate such negative effects, but its consequences on MS disease activity are yet to be ascertained. The objective of this study was to determine clinical and neuroradiologic disease activity, as well as B-cell repopulation dynamics, after implementation of extended rituximab dosing in RRMS.

**Methods:**

We conducted a prospective observational study in a specialized-care, single-center setting, including patients with RRMS participating in the COMBAT-MS and MultipleMS observational drug trials, who had received at least 2 courses of rituximab (median follow-up 4.2 years, range 0.1–8.9 years). Using Cox regression, hazard ratios (HRs) of clinical relapse and/or contrast-enhancing lesions on MRI were calculated in relation to time since last dose of rituximab.

**Results:**

A total of 3,904 dose intervals were accumulated in 718 patients and stratified into 4 intervals: <8, ≥8 to 12, ≥12 to 18, and ≥18 months. We identified 24 relapses of which 20 occurred within 8 months since previous infusion and 4 with intervals over 8 months. HRs for relapse when comparing ≥8 to 12, ≥12 to 18, and ≥18 months with <8 months since last dose were 0.28 (95% CI 0.04–2.10), 0.38 (95% CI 0.05–2.94), and 0.89 (95% CI 0.20–4.04), respectively, and thus nonsignificant. Neuroradiologic outcomes mirrored relapse rates. Dynamics of total B-cell reconstitution varied considerably, but median total B-cell counts reached lower level of normal after 12 months and median memory B-cell counts after 16 months.

**Discussion:**

In this prospective cohort of rituximab-treated patients with RRMS exposed to extended dosing intervals, we could not detect a relation between clinical or neuroradiologic disease activity and time since last infusion. Total B- and memory B-cell repopulation kinetics varied considerably. These findings, relevant for assessing risk-mitigation strategies with anti-CD20 therapies in RRMS, suggest that relapse risk remains low with extended infusion intervals. Further studies are needed to investigate the relation between B-cell repopulation dynamics and adverse event risks associated with B-cell depletion.

Multiple sclerosis (MS) is a chronic, inflammatory, demyelinating disease of the CNS, affecting 2.8 million people worldwide.^1^ Most patients initially present with acute/subacute episodes of neurologic deficit, with variable degree of reversibility, followed by a period of clinical stability, thus classified as relapsing-remitting MS (RRMS). Accumulating evidence demonstrates that B cell–depleting therapies are associated with strong suppression of RRMS inflammatory disease activity.^2-4^ So far, 2 anti-CD20 monoclonal antibodies, ocrelizumab and ofatumumab, have been approved for use in RRMS in the United States and European Union. In addition, rituximab, an older chimeric monoclonal antibody approved for rheumatoid arthritis and other indications, is increasingly being used off-label in some countries, including Sweden. The safety profile of anti-CD20 therapies in RRMS comprises an increased infection risk,^5^ including worsened COVID-19 outcomes.^6-8^ Furthermore, anti-CD20s blunt humoral responses to vaccinations,^9^ including for SARS-CoV-2.^10,11^ Collectively, these data warrant studies exploring risk-mitigation strategies to ensure an optimized benefit-risk balance for patients with RRMS treated with anti-CD20s. So far, however, such efforts comprise relatively small-sized studies with limited dosing interval prolongation and short observation time.^12,13^ Larger real-world cohort studies with sufficiently long observation time to determine time to normalization of B-cell levels and possible relation to disease activity and risk of adverse events are thus lacking. Considering an emerging safety signal regarding infections already before the COVID-19 pandemic, a pragmatic anti-CD20 dose extension program was initiated at the Academic Specialist Center in the fall of 2018 and further extended with the pandemic outbreak in 2020. The aims of this study were to determine whether prolonged rituximab dosing intervals increase the risk of RRMS disease activity and to determine B-cell repopulation dynamics in relation to infusions.

## Methods

### Study Population

**Figure 1:**
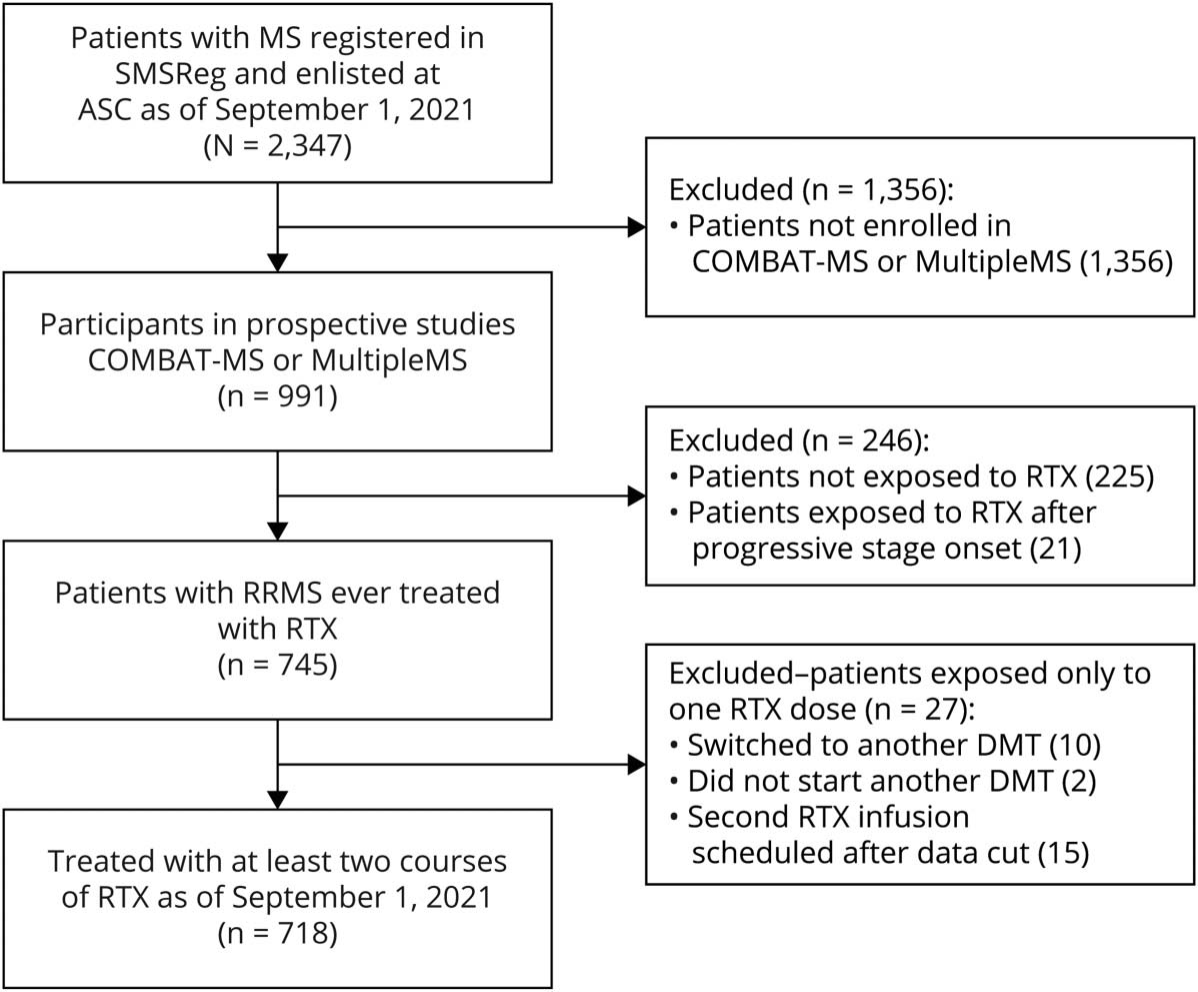
Study Population Inclusion criteria flowchart. ASC = Academic Specialist Center; DMT = disease-modifying therapy, RRMS = relapsing-remitting MS; RTX = rituximab; SMSReg = Swedish MS Registry.

The study population at the Academic Specialist Center (Stockholm, Sweden) included 718 patients with RRMS enrolled in either of 2 prospective observational drug trials, COMBAT-MS (n = 658) and MultipleMS (n = 60), who had been exposed to at least 2 doses of rituximab by September 1, 2021 (Figure 1A). The COMBAT-MS study included patients with RRMS initiating a first disease-modifying therapy (DMT) or doing a first DMT switch between January 1, 2011, and October 31, 2018. The MultipleMS study included newly diagnosed, treatment-naive patients with RRMS who initiated a first DMT between April 1, 2018, and September 28, 2020. Data regarding demographics, disease, and treatment history were extracted from the Swedish MS registry. Data on total B-cell count and percentage of B cells subpopulations were extracted from medical records from January 1, 2018, to September 1, 2021.

### Standard Protocol Approvals, Registrations, and Patient Consents

COMBAT-MS, EudraCT: 2016-003587-39, Clinicaltrials.gov identifier: NCT03193866; MultipleMS, EudraCT: 2017-002634-24. Written informed consent was obtained from all study participants (Stockholm Regional Ethics Board, no. 2017/32-31/4 and 2017/1323-31).

### Follow-up, Outcomes, and Covariates

Patients were followed annually with assessment of Expanded Disability Status Scale (EDSS), relapse history, MRI, and acute contacts when needed. The MRI protocol has been published previously and considers contrast administration for follow-up scans as optional in case of a stable patient, leaving the decision to the treating neurologist.^14^ COMBAT-MS SMSReg data have been validated against medical records for completeness, and both studies are continuously monitored for data completeness.^15^ The outcomes in the main analysis were clinical relapses and brain or spinal cord contrast-enhancing lesions (CELs). Because annual MRI scans were not synchronized with rituximab infusions, it was not possible to attribute the occurrence of new or enlarged T2 lesions to a specific infusion interval; hence, data are presented for each participant according to the longest interval ever experienced.

The effect of dose interval extension was assessed by comparing relapse rates during <8, ≥8 to 12, ≥12 to 18, and ≥18 months since last infusion. Treatment interval was analyzed as a time-varying covariate. As a result, treatment intervals longer than 8 months were spilt in more than 1 category, where for example a 14 months interval since last rituximab infusion contributed data to the <8, ≥8 and ≥12 months' time bands. The interval after the first rituximab infusion (or after the first 2 infusions if <90 days apart) was excluded from the analysis to avoid the effect of residual disease activity early after treatment start (eFigure 1, http://links.lww.com/NXI/A770).

The following were considered potential confounders: sex, age at infusion, EDSS at infusion, number of previous rituximab doses, number of clinical relapses in the year before rituximab start, number of brain MRI T2 lesions at rituximab start (categorized as 0, 1–9, 10–20, and >20), and previous DMTs, classified as none, moderately effective (injectables, dimethyl fumarate, teriflunomide, or daclizumab), highly effective (fingolimod, natalizumab, or ocrelizumab), and others (unspecified).

### B-Cell Data

Total B-cell (CD3^+^CD19^+^) levels were assessed by flow cytometry before each rituximab infusion, as per clinical routine, at the Department of Clinical Immunology, Karolinska University Hospital. B memory cell (CD3^+^CD19^+^CD27+immunoglobulin D (IgD)-, CD27+IgD+, and CD27−IgD−) percentages were determined in patients with detectable B cells and converted to absolute numbers using the extracted data. B-cell data were assessed in relation to time since last infusion (in months). In instances where multiple measurements had been performed in the same treatment interval, only the last assessment was included. B-cell samples were classified as depleted if below the detection level (10 cells/μL for total B and 0.05 cells/μL for total and CD27+IgD− memory B), partially repleted if above the detection limit, but below lower limit of normal (LLN, 80 cells/μL for total B, 15.2 cells/μL for total memory B, and 5.6 cells/μL for CD27+IgD− memory B), and completely repleted if equal or above LLN.

### Statistical Analysis

Cox proportional hazard regression models were used to calculate hazard ratios (HRs) and corresponding 95% CIs of clinical relapse or CELs in relation to rituximab dose intervals. Time since disease onset was the underlying time scale in all models; thus, HRs were compared across patients with the same disease duration. Study entry occurred at each rituximab infusion, with left truncation of follow-up time to avoid immortal time bias (i.e., we used the counting process approach, with a start and a stop defining each patient's each interval). Every time-to-censoring/outcome after a rituximab dose was subsequently split in the aforementioned time bands (<8, ≥8 to 12, ≥12 to 18, and ≥18 months), and HRs were calculated for ≥8 to 12, ≥12 to 18, and ≥18 months since last dose, using <8 months as reference (for further details, see eMethods, http://links.lww.com/NXI/A770). A sandwich estimator was used to account for exposure of the same patient to multiple rituximab dosing intervals. Models were separately analyzed as crude and adjusted for confounders (listed above). Censoring events were a subsequent rituximab infusion, conversion to secondary progressive MS (SPMS), emigration, death, or September 1, 2021, whichever came first. In the main analysis, we did not include date of switch to a different DMT as a censoring event in keeping with the hypothesis of a long-lasting effect of B-cell depletion and to maximize sensitivity for relapses. However, DMT switch (33 patients out of 718; 4.6%) was considered an additional censoring event in a sensitivity analysis to restrict the analysis time to exclusive exposure to rituximab. Negative binomial regression models were used to calculate repletion rate ratios (RRs) for total B- and memory B-cell counts per microliter in relation to months since last infusion. Both total B/memory B-cell counts per microliter and months since last rituximab infusion were regarded as numerical, continuous variables in the regression model. Stratification of the regression model by sex, age, body mass index (BMI), disease duration, and number of previous rituximab doses was performed to uncover possible effect modifiers, with continuous variables transformed in binary variables taking the approximate median value as splitting point. With this approach, age was categorized as <40 and ≥40 years, BMI as <24 and ≥24 kg/m^2^, disease duration as <8 and ≥8 years since disease onset, and number of previous rituximab doses as 1–3 or ≥4. Analyses were conducted with STATA/BE software, 17.0.

### Data Availability

Deidentified or aggregated data will be shared with qualified investigators on reasonable request and pending a relevant data transfer agreement, in compliance to European legislation.

## Results

### Study Participants

We identified 718 unique patients with RRMS enrolled in either COMBAT-MS or MultipleMS studies who had been exposed to at least 2 courses of rituximab treatment, for a total of 4622 treatment episodes (Figure 1A and eFigure 2a, http://links.lww.com/NXI/A770). Patients were followed from each rituximab infusion until subsequent infusion, occurrence of clinical relapse or CELs, emigration (n = 1), death (n = 3), transition to SPMS (n = 19), or end of follow-up, whichever came first. After exclusion of the first treatment course, these individuals had accumulated 3,904 rituximab dose intervals, for a median follow-up of 4.2 years (interquartile range 2.7–5.6 years). The baseline characteristics of study participants are summarized in Table 1.

**Table 1:**
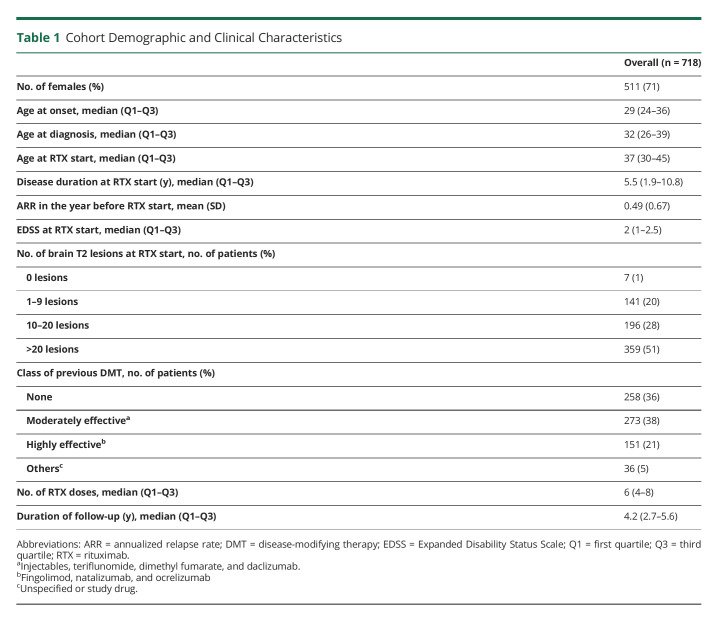
Cohort Demographic and Clinical Characteristics

The infusion-to-outcome/censoring intervals were distributed as follows: 2,577 intervals (66%) <8 months, 585 (15%) ≥8–12 months, 323 (8%) ≥12–18 months, and 419 (11%) ≥18 months. Importantly, 95% of patients (n = 683) underwent at least 1 interval extension (≥8 months), with 87% (n = 628) and 56% (n = 403) exposed to an interval of ≥12 months and ≥18 months, respectively. Of the remaining 35 participants, the main reasons for remaining on a <8 months treatment schedule were (1) censoring event before implementation of extension protocol, n = 16 (due to conversion to SPMS, n = 15, or death, n = 1); (2) receiving a second course of rituximab after January 1, 2021 (n = 10), consequently too early to have been exposed to dose interval extension; and (3) unspecified reasons (n = 9), likely involving personal preferences of the treating neurologist and/or the patient.

### Clinical Relapse Occurrence and Relation With Time Since Last Rituximab Infusion

During follow-up, a total of 24 relapses were recorded (eFigure 2b, http://links.lww.com/NXI/A770) with the annualized relapse rate dropping from a mean of 0.49 (95% CI 0.44–0.54) in the year before rituximab start to 0.03 (95% CI 0.02–0.05) in the first treatment year and ≤0.01 onward, up to 10 years of follow-up (eFigure 2c, http://links.lww.com/NXI/A770).

We subsequently determined whether longer time since last rituximab infusion was associated with increased risk of a clinical relapse. HRs compared with <8 months since last infusion were 0.28 (95% CI 0.04–2.10), 0.38 (95% CI 0.05–2.94), and 0.89 (95% CI 0.20–4.04) for ≥8 to 12, ≥12 to 18, and ≥18 months, respectively (Table 2). Adjustment for potential confounders did not substantially divert from the crude model. Adjusted HRs for ≥8 to 12, ≥12 to 18, and ≥18 months compared with <8 months since last infusion were 0.30 (95% CI 0.04–2.32), 0.42 (95% CI 0.05–3.23), and 0.85 (95% CI 0.18–3.92), respectively.

**Table 2:**
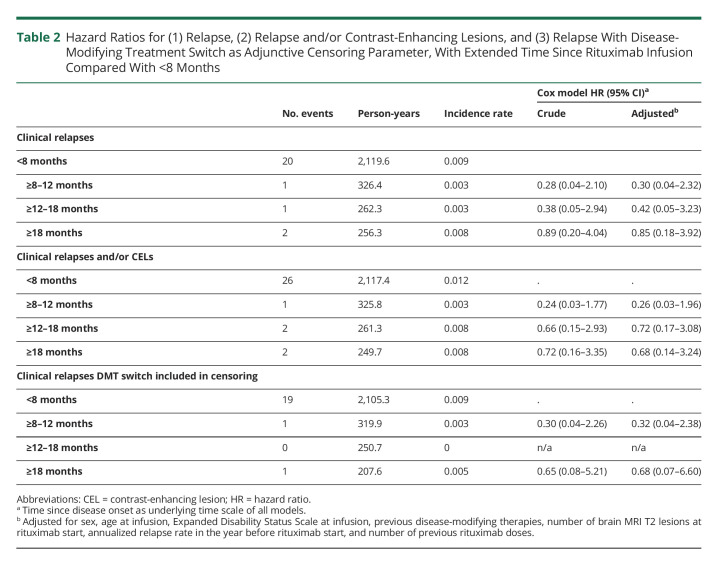
Hazard Ratios for (1) Relapse, (2) Relapse and/or Contrast-Enhancing Lesions, and (3) Relapse With Disease-Modifying Treatment Switch as Adjunctive Censoring Parameter, With Extended Time Since Rituximab Infusion Compared With <8 Months

When considering date of switch to a different DMT as adjunctive censoring parameter, 3 relapses were censored, leaving a total of 21 clinical relapses. In this setting, adjusted HRs compared with <8 months resulted in 0.32 (95% CI 0.04–2.38) for ≥8–12 months and 0.68 (95% CI 0.07–6.6) for >18 months since last rituximab dose, whereas HR for ≥12–18 months could not be calculated due to the absence of events (Table 2). Overall, these point estimates suggest a similar or lower risk of relapse with transition from a regular to an extended dosing interval regimen.

### Inclusion of Contrast-Enhancing Lesions as Outcome

Neuroradiologic assessments conducted with administration of contrast agent (1,370 brain or spinal cord MRI scans out of 3,075 total scans, 44.6%, resulting in 548 patients with at least 1 follow-up scan with contrast administration) were evaluated. Among these, 11 scans revealed CEL events, 4 of which were performed in the same treatment interval of a registered clinical relapse. When considering both relapse and/or CELs, 31 disease activity events were recorded, with HRs of 0.24 (95% CI 0.03–1.77), 0.66 (95% CI 0.15–2.93), and 0.72 (95% CI 0.16–3.35) for ≥8 to 12, ≥12 to 18, and ≥18 months, respectively, compared with <8 months since last infusion in the crude model. Similar to the previous analysis considering only clinical relapses, adjustment for potential confounders barely altered the associations. HRs were 0.26 (95% CI 0.03–1.96), 0.72 (95% CI 0.17–3.08), and 0.68 (95% CI 0.14–3.24) for ≥8 to 12, ≥12 to 18, and ≥18 months since last dose, respectively (Table 2).

**Figure 2:**
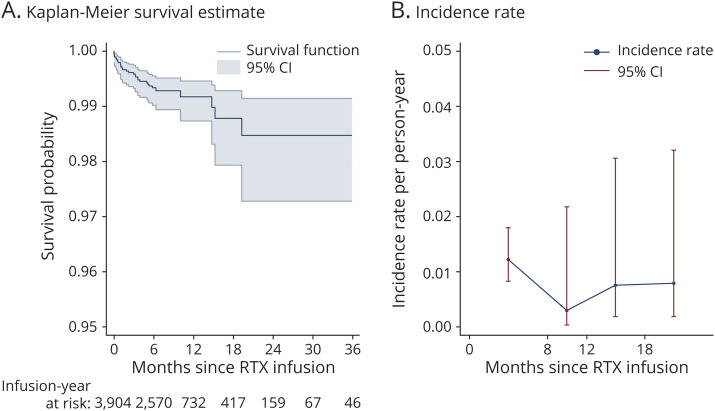
Risk of Clinical Relapse and/or Contrast-Enhancing Lesion Occurrence in Relation to Time Since Last Rituximab Infusion (A) Kaplan-Meier curve of event-free time since last rituximab infusion. (B) Incidence rate of clinical relapse and/or CELs at <8 months, ≥8 to 12, ≥12 to 18, and ≥18 months since last rituximab infusion. CEL = contrast-enhancing lesion; RTX = rituximab.

Overall, the risk of an adverse efficacy outcome did not noticeably change up to 3 years after rituximab infusion, with an event-free survival rate of 0.98 (95% CI 0.97–0.99) (Figure 2A). In the same manner, the incidence rate for relapse and/or CELs remained stable across the 4 time bands (Table 2 and Figure 2B).

### New or Enlarging T2 Lesions on MRI Scans

Forty-eight MRI scans in 44 patients (6.9% of 636 patients with valid follow-up and reference MRI scans) displayed new or enlarging T2 lesions. As yearly scans were not synchronized with rituximab infusions, the time period covered between follow-up and reference MRI scans did not match a specific treatment interval, so data are reported per individual instead of per dosing interval, with stratification for the longest treatment interval ever experienced when increase in T2 lesion burden was recorded. With this approach, 25 patients were in the <8 months group (out of 90 patients in this group, 27.8%), whereas 7 (out of 144, 4.8%), 4 (out of 242, 1.7%), and 8 (out of 160, 5%) were in the ≥8 to 12, ≥12 to 18, and ≥18 months groups, respectively.

### Total B-Cell Repopulation Kinetics

**Figure 3:**
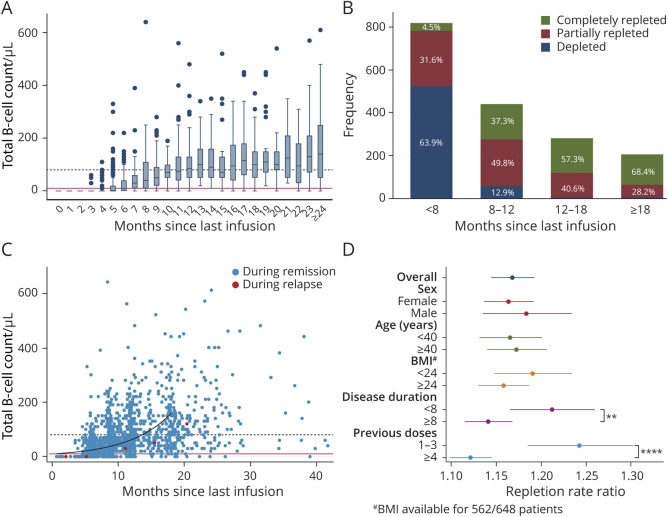
Total B-Cell Repopulation Dynamics in Relation to Time Since Last Rituximab Infusion (A) Box plot depicting distributions of B-cell count grouping samples into 1-month-time intervals since last rituximab infusion. Continuous red line: B-cell detection limit; dashed black line: LLN. (B) Total number of observations (y axis) with relative frequencies (bar labels) of depleted (<10 cells/μL), partially repleted (≥10–80 cells/μL), and completely repleted (≥80 cells/μL) B-cell counts at <8 months, ≥8 to 12, ≥12 to 18, and ≥18 months since last rituximab infusion. (C) Scatter plot of total B-cell counts in relation to time since last infusion, with samples collected within 1 month from a relapse/contrast-enhancing lesion highlighted in red. The predicted repopulation kinetic up to 18 months since last rituximab infusion according to a negative binomial regression model is depicted by the black curve. Continuous red line: B-cell detection limit; dashed black line: lower limit of normal. (D) Coefficient plot of the repletion rate ratios (RRs) for B-cell repopulation for the overall model and stratified according to sex, age (<40 and ≥40 years of age), BMI (<24 and ≥24 kg/m^2^), disease duration (<8 and ≥8 years), and number of previous rituximab doses (1–3 and ≥4). Among the analyzed potential effect modifiers, disease duration and number of previous rituximab doses affected B-cell reappearance kinetics (Wald test *p* < 0.01 for disease duration, **, and *p* < 0.0001 for rituximab doses, ****). #BMI data were available for 86.7% of the patients, 86.5% of the samples.

B-cell data were available only after January 1, 2018, resulting in 1,744 data points for a total of 2,208 infusions after this date in 648 patients (79% of the infusions; 90% of the patient cohort). The median of total B-cell counts reached detectable levels 6 months since last infusion and the LLN after 12 months (Figure 3A). However, a considerable degree of variability of total B-cell levels was observed, with variance increasing over time since last infusion and a small proportion of subjects (3.4%) remaining completely depleted even with the longest dosing intervals. When considering the same time bands used in the main analysis, we found that 63.9% of samples remained depleted in the <8 months group. In contrast, the majority of samples were partially or completely repleted in the extended-interval groups. The 2 categories combined accounted for 87.1%, 97.9%, and 96.6% of the samples in the ≥8 to 12, ≥12 to 18, and ≥18 months groups, respectively (Figure 3B). Negative binomial regression confirmed positive association between B-cell levels and months since last infusion, with a repletion RR of 1.17 (95% CI 1.14–1.19, *p* < 0.0001). In other words, the model predicted an increase in B-cell counts of 17% every month in the analyzed data set. When stratifying according to age, sex, BMI, disease duration, or number of previous rituximab doses, we did not observe any significant effect modification by sex, age, and BMI, whereas longer disease duration and higher number of previous rituximab doses were associated with a slower rate of B cell repopulation, as expressed by the RR (interaction coefficients 0.94, 95% CI 0.9–0.98 and 0.9, 95% CI 0.86–0.95 for disease duration and number of previous doses, respectively). The stratified RRs were 1.21 (95% CI 1.18–1.25) and 1.14 (95% CI 1.12–1.16) for disease duration <8 or ≥8 years, respectively, and 1.24 (95% CI 1.19–1.30) and 1.12 (95% CI 1.10–1.15) for B-cell repopulating after infusions preceded by 1–3 or ≥4 rituximab doses, respectively (Figure 3, C and D).

### Memory B-Cell Repopulation Kinetics

**Figure 4:**
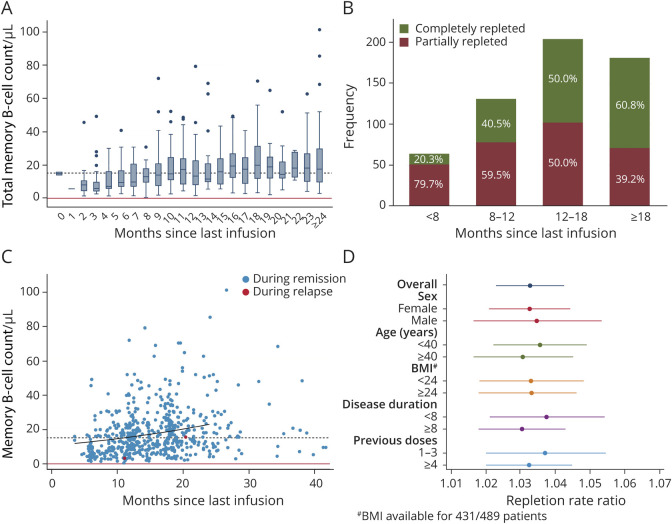
Memory B-Cell Repopulation Dynamics in Relation to Time Since Last Rituximab Infusion (A) Box plot showing distributions of memory B-cell count grouping samples into 1-month time intervals since last rituximab infusion. Continuous red line: memory B-cell detection limit; dashed black line: lower limit of normal. (B) Total number of observations (y axis) with relative frequencies (bar labels) of depleted (<0.05 cells/μL), partially repleted (≥0.05–15.2 cells/μL), and completely repleted (≥15.2 cells/μL) levels of memory B cells at <8 months, ≥8 to 12, ≥12 to 18, and ≥18 months since last rituximab infusion. (C) Scatter plot of memory B-cell counts in relation to time since last infusion, with samples collected within 1 month from a relapse/contrast-enhancing lesion highlighted in red. The repopulation kinetic predicted by a negative binomial regression model up to 24 months since last rituximab infusion is shown by the black curve. Continuous red line: memory B-cell detection limit; dashed black line: lower limit of normal. (D) Coefficient plot of the repletion rate ratios (RRs) for memory B-cell repopulation for the overall model and stratified according to sex, age, BMI, disease duration, and number of previous rituximab doses. None of the models showed any effect of the analyzed covariates on memory B-cell repletion rates. ^#^BMI data were available for 88.1% of the patients, 86.9% of the samples. BMI = body mass index

**Figure 5:**
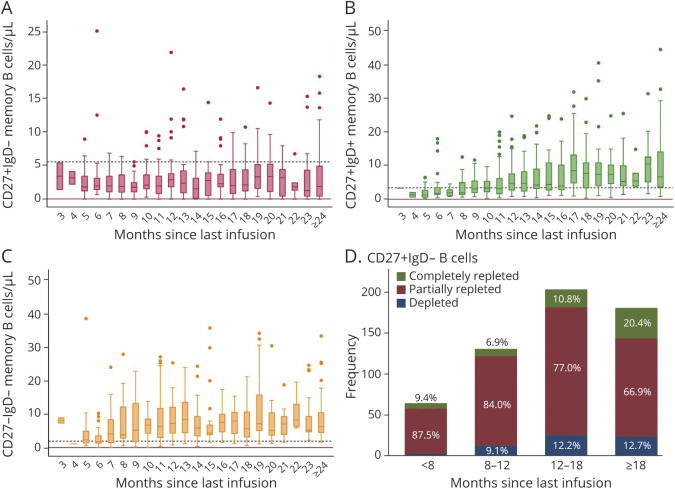
Memory B Subpopulation Repopulation Dynamics (A–C) Box plot showing distributions of the 3 subsets of memory B cell: CD27+IgD− (A), CD27+IgD+ or double positive (DP, B), and CD27−IgD− or double negative (DN, C). Samples were grouped into 1-month time intervals since last rituximab infusion. Continuous red line: memory B-cell detection limit (0.05 cells/μL for all subsets); dashed black line: lower limit of normal (5.6 cells/μL for CD27+IgD− cells, 3.44 cells/μL for DP cells and 1.92 cells/μL for DN cells). (D) Total number of observations (y axis) with relative frequencies (bar labels) of depleted (<0.05 cells/μL), partially repleted (≥0.05–5.6 cells/μL), and completely repleted (≥5.6 cells/μL) levels of CD27+IgD− memory B cells at <8 months, ≥8 to 12, ≥12 to 18, and ≥18 months since last rituximab infusion.

Memory B-cell data were available only for samples with detectable B cells. In this subgroup of 1,157 samples, 580 samples (46%) also included B-cell subpopulation analysis, for a total of 489 patients. Compared with total B-cell levels, memory B cells showed a slower repopulation kinetic: the median of memory B-cell counts, categorized in 1-month time bands, was steadily above the limit of detection but reached LLN only after 16 months (Figure 4A). Thus, memory B cells were partially repleted in the majority of samples in the first 2 time bands (79.7% and 59.5% for the <8 and ≥8–12 months, respectively), whereas most samples (60.8%) were normalized after 18 months since last infusion (Figure 4B). Accordingly, when applying a negative binomial regression model, the repletion rate ratio was 1.03 (95% CI 1.02–1.04, Figure 4C). No effect modification was observed stratifying samples according to age, sex, BMI, disease duration, or number of previous rituximab doses (Figure 4D). When considering separately the 3 subsets composing memory B cells (CD27+IgD−, CD27+IgD+ or double positive, DP, and CD27−IgD− or double negative, DN), we noted different repopulation kinetics, with LLN reached in 12 and 7 months for DP and DN subsets, respectively (Figure 5, A and B), whereas median levels of CD27+IgD− memory subpopulation persistently remained below LLN for up to 24 months (Figure 5C). As a result, this subset was only partially repleted in the majority of samples up to 24 months since last rituximab infusion: 87.5%, 84%, 77%, and 66.9% partially repleted samples for <8, ≥8 to 12, ≥12 to 18, and ≥18 months, respectively (Figure 5D).

## Discussion

We here related inflammatory disease activity to time since last rituximab infusion in a large cohort of patients with RRMS exposed to dose interval extension with long prospective follow-up. Alike the ocrelizumab label, the Swedish MS Society recommends rituximab dosing (500 mg) every 6 months. Because of an emerging signal for increased infection rate over time,^5^ from October 2018, the Academic Specialist Center changed guidelines to recommend dose extension to 12 months after the fifth infusion for patients with stable disease. With the start of the COVID-19 pandemic in March 2020, infusion intervals were further extended to 12–24 months or more from the second infusion regardless of treatment duration, a decision also influenced by accumulating evidence suggesting an adverse effect on COVID-19 outcomes.^6,7^

We previously reported the absence of rebound disease activity in patients with RRMS who, for various reasons, had stopped rituximab for more than 12 months.^16^ Similar results were reported for patients with RRMS exposed to low dose (<1000 mg yearly) or stopping treatment in another Swedish study.^17^ Furthermore, based on data from the phase II trial of ocrelizumab, which included a safety follow-up of about 12 months without treatment, it has been suggested that treatment intervals could be extended well beyond the regular 6-month interval.^18^ More recently, a retrospective observational study did not detect signs of disease activity rebound with extension of ocrelizumab intervals by 4 weeks or more.^12^ Finally, a Dutch prospective study implemented a personalized dosing interval of ocrelizumab based on B cell counts.^13^ In this study B-cell levels were assessed monthly after 6 months from the last infusion with retreatment with counts ≥10 cells/µL, which occurred at a median of 34 weeks since last infusion. However, all these studies were relatively small and had considerably shorter follow-up periods than included here, with a median structured prospective follow-up of 4.2 years (interquartile range 2.7–5.6 years).

Of interest, we could not detect an increased risk of clinical relapses and/or CELs for any of time bands analyzed, i.e., ≥8 to 12, ≥12 to 18, and ≥18 months, with point estimates of relapse risk remaining remarkably stable. Although the power to detect a difference was limited due to the limited number of relapse/CEL events, our observations suggest that both relapse risk and neuroradiologic disease activity remain low well beyond regular infusion intervals. The fact that HRs with extended dosing intervals in all cases were below 1 compared with the regular interval is reassuring, but likely can be influenced by overall treatment duration and a small number of subjects switching treatment or being held on shorter infusion intervals. It is important to note, however, that a substantial enrichment of stable patients in the longer time bands is unlikely given the prospective study design and the high proportion of participants being exposed to extended intervals. Taken together, these results are valuable for designing further, sufficiently powered confirmatory studies, but meanwhile provide preliminary results indicating a viable approach for improving the benefit-risk balance with B cell–depleting DMTs. This is especially relevant in context of increased susceptibility to infections, lowered immunoglobulin levels, scheduling of vaccinations, or planning of pregnancy.^5,10,19,20^

The striking efficacy of anti-CD20 therapies underscores the role of B cells in MS disease pathogenesis. Experiences across a wide spectrum of autoimmune conditions indicate numerous ways by which B cells can contribute to disease, which also depends on the type of disease process.^21^ In MS, B cells have been shown to produce cytokines with a presumed role in supporting CNS inflammation, with supporting functional data obtained in experimental autoimmune encephalitis, an animal model of MS.^22,23^ Notably, however, antigen-specific memory B-cell clones have also been shown to activate memory T cells with encephalitogenic features in subjects with RRMS ex vivo.^24^ The possible implication of this observation is that disease-driving cells mainly belong to the memory B-cell subtype, alike what has been suggested for neuromyelitis optica, where monitoring of memory B-cell subset has been suggested to be useful to determine anti-CD20 infusion intervals.^25,26^ However, it is important to acknowledge that clinical effectiveness of extended dosing intervals cannot readily be extrapolated due to different pathomechanisms, whereas it is more likely that B-cell repopulation kinetics will be more similar across the 2 conditions. However, in spite of increasing use of anti-CD20 therapies in MS, there is only limited information on the kinetics of B-cell repopulation after depletion and if there is any relation with return of disease activity. Insights regarding different memory B-cell subsets are also lacking. To this end, we analyzed total B-cell and memory B-cell counts determined in clinical routine for our cohort. Although limited by a certain irregularity of assessments in this real-world setting, our data, nevertheless, demonstrate a considerable variability in reconstitution kinetics of total B-cell numbers, where median counts were normalized after 12 months, whereas memory B cells remained below LLN for up 16 months since last dose. In the prediction model we used, based on negative binomial regression, total B-cell counts increased by 17% every month, with disease duration and number of previous rituximab doses negatively affecting repopulation rates, although results should be interpreted with some caution due to the variability in timing of sampling. Regarding memory B cells, the repletion rate was much slower, with a monthly increase of only 3%. When considering different memory B-cell subsets, CD27−IgD− were the first to repopulate, followed by CD27+IgD+, whereas median levels of CD27+IgD− remained consistently below LLN up to 24 months since last infusion. Overall, these results might hint at different roles of memory B-cell subsets in RRMS pathogenesis. The low number of adverse efficacy events coupled with lack of systematic determination of B cells at occurrence of clinical or radiologic activity meant that a comparison between nonactive and active patients could not be performed. However, it is evident that a strong signal for return of inflammatory disease activity with repletion of B cells, including of the memory subtype, is lacking.

Apart from previously mentioned limitations relating to the real-world nature of this study, including incomplete data coverage and variability in the structure of data collection, this study also did not include volumetric MRI data or soluble biomarkers such as neurofilament light chain concentrations. Thus, we cannot exclude that dose interval extension negatively affects disease processes not reflected by relapses or accrual of focal MRI lesions, and studies exploring the effect of extended B cell–depleting treatment schedules on the progressive aspects of MS are therefore warranted.

In summary, our findings suggest that anti-CD20 dose interval extension could be considered in patients with RRMS with stable disease without incurring risk of return of inflammatory disease activity in the short to medium term, especially in case of treatment-related adverse events or when planning pregnancy. Further studies are needed to determine whether dose interval extension is also associated with a lowered risk of infection, while it has been shown that vaccination responses are improved with B-cell repopulation,^27^ in turn improving benefit-risk with anti-CD20 therapies.

## Glossary

CEL: contrast-enhancing lesion
DMT: disease-modifying therapy
EDSS: Expanded Disability Status Scale
HR: hazard ratio
LLN: lower limit of normal
MS: multiple sclerosis
RR: rate ratio
RRMS: relapsing-remitting MS
SPMS: secondary progressive MS
